# Systolic blood pressure as a predictor of transient ischemic attack/minor stroke in emergency department patients under age 80: a prospective cohort study

**DOI:** 10.1186/s12883-019-1466-4

**Published:** 2019-10-25

**Authors:** Andrew M. Penn, Nicole S. Croteau, Kristine Votova, Colin Sedgwick, Robert F. Balshaw, Shelagh B. Coutts, Melanie Penn, Kaitlin Blackwood, Maximilian B. Bibok, Viera Saly, Janka Hegedus, Amy Y. X. Yu, Charlotte Zerna, Evgenia Klourfeld, Mary L. Lesperance

**Affiliations:** 1 Stroke Rapid Assessment Unit, Island Health, Victoria, BC Canada; 2 Department of Research and Capacity Building, Island Health, 1952 Bay Street, Victoria, BC V8R1J8 Canada; 3 Department of Mathematics and Statistics, University of Victoria, Victoria, BC Canada; 4 Division of Medical Sciences, University of Victoria, Victoria, BC Canada; 5 George & Fay Yee Centre for Healthcare Innovation, University of Manitoba, Winnipeg, MB Canada; 6 Departments of Clinical Neurosciences, Radiology, and Community Health Services, Hotchkiss Brain Institute, Foothills Medical Centre, University of Calgary, Calgary, AB Canada; 7 Department of Medicine, Sunnybrook Health Sciences Centre, University of Toronto, Toronto, ON Canada

**Keywords:** Transient ischemic attack, TIA, Minor stroke, Blood pressure, Emergency medicine

## Abstract

**Background:**

Elevated blood pressure (BP) at emergency department (ED) presentation and advancing age have been associated with risk of ischemic stroke; however, the relationship between BP, age, and transient ischemic attack/minor stroke (TIA/MS) is not clear.

**Methods:**

A multi-site, prospective, observational study of 1084 ED patients screened for suspected TIA/MS (symptom onset < 24 h, NIHSS< 4) between December 2013 and April 2016. Systolic and diastolic BP measurements (SBP, DBP) were taken at ED presentation. Final diagnosis was consensus adjudication by stroke neurologists; patients were diagnosed as either TIA/MS or stroke-mimic (non-cerebrovascular conditions). Conditional inference trees were used to define age cut-points for predicting binary diagnosis (TIA/MS or stroke-mimic). Logistic regression models were used to estimate the effect of BP, age, sex, and the age-BP interaction on predicting TIA/MS diagnosis.

**Results:**

Over a 28-month period, 768 (71%) patients were diagnosed with TIA/MS: these patients were older (mean 71.6 years) and more likely to be male (58%) than stroke-mimics (61.4 years, 41%; each *p* < 0.001). TIA/MS patients had higher SBP than stroke-mimics (*p* < 0.001). DBP did not differ between the two groups (*p* = 0.191). SBP was predictive of TIA/MS diagnosis in younger patients, after accounting for age and sex; an increase of 10 mmHg systolic increased the odds of TIA/MS 18% (odds ratio [OR] 1.18, 95% CI 1.00–1.39) in patients < 60 years, and 23% (OR 1.23, 95% CI 11.12–1.35) in those 60–79 years, while not affecting the odds of TIA/MS in patients ≥80 years (OR 0.99, 95% CI 0.89–1.07).

**Conclusions:**

Raised SBP in patients younger than 80 with suspected TIA/MS may be a useful clinical indicator upon initial presentation to help increase clinicians’ suspicion of TIA/MS.

**Trial registration:**

ClinicalTrials.gov NCT03050099 (10-Feb-2017) and NCT03070067 (3-Mar-2017). Retrospectively registered.

## Background

Transient ischemic attack and minor stroke (TIA/MS) are characterized by mild focal neurological deficits, [1, 2] and occur at the lower end of the ischemic continuum [Albers]. Once believed to be benign due to its transient nature, TIA is now thought to be prodromal for stroke with approximately 5% of TIA/MS patients suffering a recurrent stroke within 90 days [3–8]. The risk of subsequent stroke following TIA/MS is front-loaded, with many events occurring in the first 24–48 h [4, 6, 9, 10]. The short-term risk of stroke following a TIA/MS event accentuates the need for early diagnosis and intervention. Neuroimaging, such as magnetic resonance imaging (MRI) and computed topography (CT) are recommended clinical investigations to diagnose TIA/MS and to identify high-risk cases [11]. Yet, not all health care centers have ready-access to imaging services, nor the capacity to image all suspected patients. Clinicians are thus required to use a combination of clinical signs and symptoms to triage TIA/MS patients for appropriate care. Symptoms of TIA/MS, however, are not unique to TIA/MS and occur frequently in low-risk, non-cerebrovascular, conditions that mimic TIA/MS in clinical presentation, such as migraine. Such stroke-mimic conditions complicate the role of clinicians when allocating neuroimaging and health care resources to suspected TIA/MS patients.

Diagnosing TIA/MS is challenging for first-contact physicians due to the high prevalence of stroke-mimic conditions [12]. A recent meta-analysis estimating the diagnostic accuracy for acute cerebrovascular events in the ED found 9% of events were missed entirely and nearly 42% of TIA cases were misdiagnosed [13]. Along with the motor and speech deficits common in the presentation of brain ischemic patients, TIA/MS patients may also present with vestibular and vision disturbances, clinical symptoms frequently associated with stroke-mimic conditions, such as vertigo and diplopia [12]. A further challenge in diagnosing TIA/MS is that many physicians are reserved in clinically suspecting TIA/MS in younger patients as the condition is more often associated with advanced age [14, 15].

Blood pressure (BP) is an objective vital sign that is readily available during any ED encounter to assist healthcare staff in deciding among differential patient pathways. The relationship between BP and ischemic stroke has been well-studied. During paramedic assessments, Systolic blood pressure (SBP) is higher in acute stroke patients relative to stroke-mimics [16] and at initial hospital presentation, elevated BP significantly distinguishes between stroke and stroke-mimic patients [17]. After hospitalization, BP tends to spontaneously decrease [18]. In contrast, the connection between BP and TIA/MS is under-studied. Most TIA studies aim to quantify the subsequent risk of stroke following TIA with a prognostic clinical tool. BP is incorporated into many of these prediction rules, including the ABCD [19], ABCD2 [6], and Canadian TIA Score [20], yet the significance of BP as a diagnostic predictor of TIA has been reported with conflicting results. Some studies suggest BP may differentiate TIA/MS and non-cerebrovascular events in the population [21], while others have shown BP not to be a significant predictor for TIA [12].

The purpose of our study is to investigate the role BP can serve in differentiating between TIA/MS and stroke-mimic patients who present at the ED less than 24 h after symptom onset. Our study objectives were (1) to assess differences in BP of TIA/MS and stroke-mimic patients at ED triage, and (2) to further assess the effect of BP for discriminating between TIA/MS and stroke-mimics after controlling for risk-factors and clinical confounders.

## Methods

### Study design and procedure

The present study is part of a larger multi-site, prospective, observational TIA biomarker study [22, 23]. The study sites include three hospital EDs (one in Alberta, and two on Vancouver Island, Canada) that refer suspected TIA/MS patients to their respective regional stroke prevention clinics. Access to the stroke clinics is through healthcare-provider referral only. The clinics provide early assessment and treatment by stroke neurology teams in an outpatient setting.

Patients with suspected TIA/MS were enrolled by study nurses in the ED, 7 days a week, over the 28-month study period December 2013 to April 2016. Enrollment inclusion criteria were: (i) patient presented to the ED with signs and symptoms of TIA/MS; (ii) NIHSS score < 4; (iii) symptom onset < 24 h; (iv) age ≥ 18 years; and (v) English speaking or translator available. Participants with isolated monocular blindness, hemorrhagic stroke (subarachnoid and intracranial), and/or who were unable to have either MRI within 7 days or CT angiography (CTA) within 24 h were excluded. All study patients received either MRI or CT/CTA, as per the study protocol.

Clinical data were obtained from the patients by stroke nurses in the ED and recorded on a standardized case report form. A variety of patient-reported disease and lifestyle risk factors were recorded. Blood pressure was measured at ED triage by non-study, hospital staff prior to any imaging investigations; hence, BP measurements were naturally blinded to later imaging results. Patients were either prone or sitting during BP measurements. All study participants were referred as per standard of care by the attending ED physician for follow-up specialist services at the outpatient TIA clinics where they received a full neurological assessment. Study procedures included MRI or CTA imaging in the ED prior to outpatient referral, a blood sample, and comprehensive clinical information obtained by study stroke-nurses in the ED. Ninety-day outcomes were assessed based on patient (or proxy) self-report during a study-coordinator led follow-up by phone or hospital record chart review to ascertain stroke recurrence or death after initial event.

Institutional approval to conduct the research was provided by each hospital’s Ethics Review Board. All patients provided written informed consent. The study adhered to CONSORT reporting guidelines.

### Definitions

Diagnosis was made by stroke neurologists during the patient’s clinic visit and was supported by MRI and/or CT/CTA imaging results. Cases were adjudicated by stroke neurologists in consensus. For this study’s purpose, stroke-mimics were defined as those patients with clinical presentation inconsistent with TIA/MS and negative MRI imaging results. We conceptualized diagnoses of TIA and minor-stroke as both existing on the lower-end of the brain ischemia continuum [1, 24] and treated the conditions as equivalent. This decision was motivated by the recognition that treatments for both conditions are identical (i.e., prophylactic risk factor management), and that within the first 24 h of symptom onset no clinical distinction between the conditions is discernable (i.e., deficits persisting past 24 h in the case of minor-stroke) [11]. TIA was defined as a “brief episode of neurologic dysfunction caused by focal brain or retinal ischemia, with clinical symptoms typically lasting less than one hour, and without evidence of acute infarction … [t] he corollary is that persistent clinical signs or characteristic imaging abnormalities define infarction — that is, stroke” (p. 1715) [25], with minor-stroke defined as stroke with an NIHSS score [26].

The modified TOAST (Trial of Org 10,172 in Acute Stroke Treatment) criteria was used to classify TIA/MS diagnoses into five subtypes: (i) large-artery atherosclerosis (LAA), (ii) cardioembolic (CE), (iii) small-vessel occlusion (SVO), (iv) other determined etiology, and (v) cryptogenic (i.e. undetermined etiology). Where two or more causative subtypes were identified, a sixth group, competing etiology, was created for analysis. The classification system was applied using clinical features and the results of ancillary diagnostic studies and has been shown to have good inter-rater reliability [27].

### Statistical analysis

Categorical data were reported as counts and proportions; continuous variables were reported as means and standard errors (SE) or medians and interquartile ranges (IQR), as appropriate. Differences in the distribution of baseline characteristics between patient groups were assessed using the Pearson’s chi-square test of homogeneity, Welch’s t- [28], and Mann-Whitney tests [29]. Differences in BP between the diagnostic groups were analyzed by one-way ANOVA followed by Tukey’s honest significant difference test [30] which adjusts for multiple comparisons. An alpha level of 0.05 was used as a critical value for all statistical tests.

To assess the effect of BP in discriminating TIA/MS and stroke-mimic patients, and to investigate potential interaction terms, we first fitted conditional inference trees with six pre-specified covariates: BP (systolic or diastolic, measured in mmHg), age (decades), sex (female/male), hypertension (no/yes), diabetes (no/yes), and anxiety (no/yes) as a reported symptom.

The tree algorithm recursively performs univariate splits of the data based on values of a set of covariates. At each step, the algorithm tests a global null hypothesis of independence between the response and any of the covariates. If the hypothesis cannot be rejected, the algorithm stops; otherwise, it selects the covariate most strongly associated with the response and finds an optimal binary split in the given covariate using a permutation test framework [31].

A logistic regression model was then fit using the same set of covariates used in fitting the tree model in addition to an age-BP interaction term; however, the age variable was replaced by the categorical age-groups as defined by the tree model. From this full model, a reduced model was then found by backward elimination (AIC criterion) while not allowing BP to be dropped from the model. The model selection procedure was internally validated using bootstrap resampling (B = 200), and we report the frequency of variable selection.

Analyses were performed in R v3.3.1 using the packages party v1.2–3, tableone v0.8.1, and rms v5.1–1 [31–33].

## Results

### Study population

During the study period 1120 participants were enrolled. After protocol violations (*n* = 30) and missing BP data (*n* = 6) were removed, our final analysis sample consisted of 1084 patients. Patient demographics are shown in Table 1. There were 768 (71%) patients diagnosed with TIA/MS and 316 (29%) diagnosed as stroke-mimic. The stroke-mimic diagnoses were as follows: migraine (28%), other (19%), peripheral vestibulpoathy (14%), seizure (10%), psychogenic/anxiety/hyperventilation (9%), syncope (5%), peripheral neuropathy/radiculopathy (5%), drop attack (3%), neurodegenerative disease (2%), tumor (2%), constitutional (1%), multiple sclerosis (1%), cranial neuropathy (1%), myelopathy (< 1%), and trauma (< 1%). TIA/MS patients were older and more likely to be male (each *p* < 0.001). The median time from reported symptom onset to ED presentation was comparable among the two groups (*p* = 0.802). Compared to their TIA/MS counterparts, stroke-mimics had fewer clinical investigations (e.g. MRI, CT, or CTA). The distribution of stroke etiology among the TIA/MS patients according to TOAST was as follows: 285 cryptogenic (37.1%), 124 CE (16.1%), 122 LAA (15.9%), 77 SVO (10.0%), 22 other known causes (2.9%), 34 competing etiology (4.4%). Patients with incomplete TOAST evaluations (104; 13.5%) were excluded from the TOAST-specific analysis.

**Table 1:** Baseline patient characteristics

Variable	All patients (*N* = 1084)	TIA/minor stroke (*N* = 768; 70.8%)	Stroke-mimic (*N* = 316; 29.2%)	p*
Demographics				
Age, mean (SD)	68.7 (15.5)	71.6 (13.7)	61.4 (17.0)	< 0.001
Sex, male (%)	573 (52.9)	442 (57.6)	131 (41.5)	< 0.001
Symptom onset to ED presentation, hours, median [IQR]	2.3 [1.3, 4.9]	2.3 [1.3, 4.9]	2.3 [1.3, 4.6]	0.862
Time of ED presentation				0.317
(midnight—4 am]	70 (6.5)	51 (6.6)	19 (6.0)	
(4 am—8 am]	85 (7.8)	61 (7.9)	24 (7.6)	
(8 am—noon]	390 (36.0)	267 (34.8)	123 (38.9)	
(noon—4 pm]	293 (27.0)	207 (27.0)	86 (27.2)	
(4 pm—8 pm]	144 (13.3)	100 (13.0)	44 (13.9)	
(8 pm—midnight]	102 (9.4)	82 (10.7)	20 (6.3)	
Anxiety/Panic Feeling (%)	67 (6.2)	44 (5.7)	23 (7.3)	0.410
Investigations (%)				
MRI	999 (92.2)	720 (93.8)	279 (88.3)	0.004
CTA	884 (81.5)	660 (85.9)	224 (70.9)	< 0.001
CT	1014 (93.5)	737 (96.0)	277 (87.7)	< 0.001
Medical History (%)				
Diabetes	193 (17.8)	143 (18.6)	50 (15.8)	0.314
Hypertension	620 (57.2)	471 (61.3)	149 (47.2)	< 0.001
Medications (%)				
Statin for at least the last 30 days	349 (32.2)	265 (34.6)	84 (26.6)	0.013
Antiplatelets for at least the last 7 days	386 (35.6)	296 (38.6)	90 (28.5)	0.002
Beta blocker	207 (19.1)	166 (21.6)	41 (13.0)	0.001
Calcium blocker	150 (13.8)	115 (15.0)	35 (11.1)	0.111
Diuretic	188 (17.3)	146 (19.0)	42 (13.3)	0.030
ACE inhibitor	262 (24.2)	197 (25.7)	65 (20.6)	0.090
Angiotensin II receptor antagonist	178 (16.4)	137 (17.8)	41 (13.0)	0.061
Vitamin K antagonist	37 (3.4)	30 (3.9)	7 (2.2)	0.225
Novel anticoagulant	46 (4.2)	39 (5.1)	7 (2.2)	0.050
90-day Outcomes (%)				0.003
Death	23 (2.1)	16 (2.1)	7 (2.2)	
Stroke	33 (3.0)	31 (4.0)	2 (0.6)	
Lost to follow-up	55 (5.1)	31 (4.0)	24 (7.6)	
No event	973 (89.8)	690 (89.8)	283 (89.6)	
TOAST (%)				
Cardioembolic	124 (11.4)	124 (16.1)		
Cryptogenic	285 (26.3)	285 (37.1)		
Large-artery atherosclerosis	122 (11.3)	112 (15.9)		
Small-vessel occlusion	77 (7.1)	77 (10.0)		
Other known etiologies	22 (2.0)	22 (2.9)		
Competing etiologies	34 (3.1)	34 (4.4)		

Medication data for statin, antiplatelets, and vitamin K antagonist missing for 1 case; TOAST subtype unavailable for 104 cases due to incomplete evaluation.

*CT* computed tomography, *CTA* computed tomography angiography, *ED* emergency department, *MRI* magnetic resonance imaging, *SD* standard deviation.

*p-values computed from Pearson’s chi-square test of homogeneity for categorical variables, Welch’s t-test and Mann-Whitney test for continuous variables reported with mean and median, respectively

### BP at ED triage in TIA/MS and stroke-mimics

**Fig. 1:**
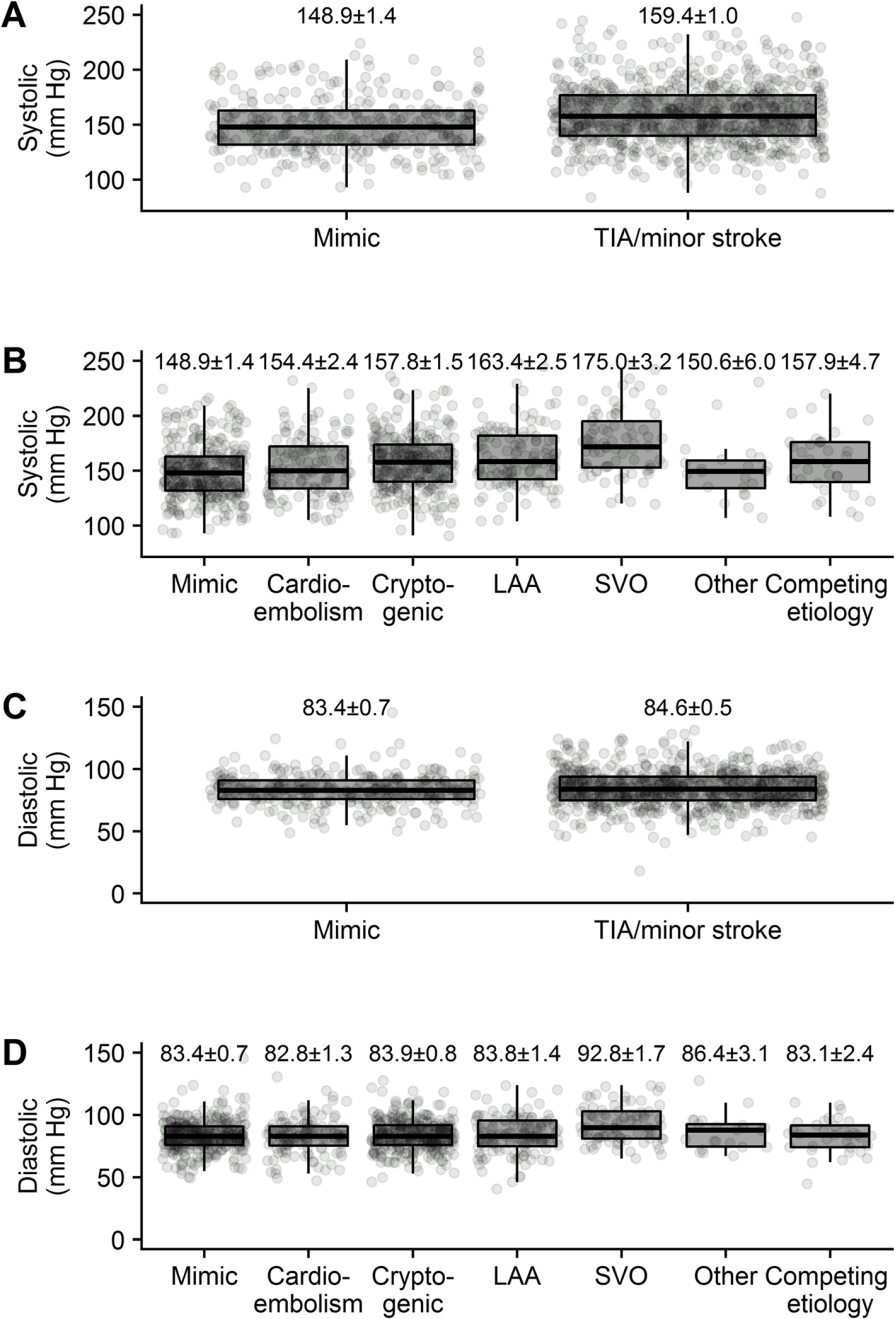
Relationship between systolic blood pressure and (**a)** final diagnosis, **b** TOAST classification, and between diastolic blood pressure and (**c**) final diagnosis, **d** TOAST classification. Blood pressure sample means ± standard errors are plotted

Figure 1 shows the distribution of BP among the patient groups. At ED triage, mean SBP was higher among TIA/MS patients (mean ± se: 159.4 ± 1.0 mmHg) compared to stroke-mimics (148.9 ± 1.4 mmHg, *p* < 0.001). No difference in DBP was detected between the two groups (*p* = 0.191).

When divided into groups based on TOAST criteria and including a stroke-mimic category, differences in SBP and DBP were seen across TOAST classifications (both *p* < 0.001). Patients with SVO had higher SBP (175.0 ± 3.2 mmHg) than every other group: stroke-mimic (148.9 ± 1.4 mmHg, *p* < 0.001), CE (154.4 ± 2.4 mmHg, *p* < 0.001), cryptogenic (157.8 ± 1.5 mmHg, *p* < 0.001), LAA (163.4 ± 2.5 mmHg, *p* = 0.041), other (150.6 ± 6.0 mmHg, *p* = 0.002), and competing etiologies (157.9 ± 4.7 mmHg, *p* = 0.027). Stroke-mimics had lower SBP compared to the LAA (*p* < 0.001) and cryptogenic (*p* = 0.001) patients. A similar trend was seen with DBP—SVO patients had higher DBP (92.8 ± 1.7 mmHg) than stroke-mimic (83.4 ± 0.7 mmHg, *p* < 0.001), CE (82.8 ± 1.3 mmHg, *p* < 0.001), cryptogenic (83.9 ± 0.8 mmHg, *p* < 0.001), LAA (83.8 ± 1.4 mmHg, *p* < 0.001), and competing etiologies (83.1 ± 2.4 mmHg, *p* = 0.01). Based on these findings, we conclude that SVO strokes have significantly higher SBP and DBP compared to most other etiologies.

### Age-BP interaction and age categorization

**Fig. 2:**
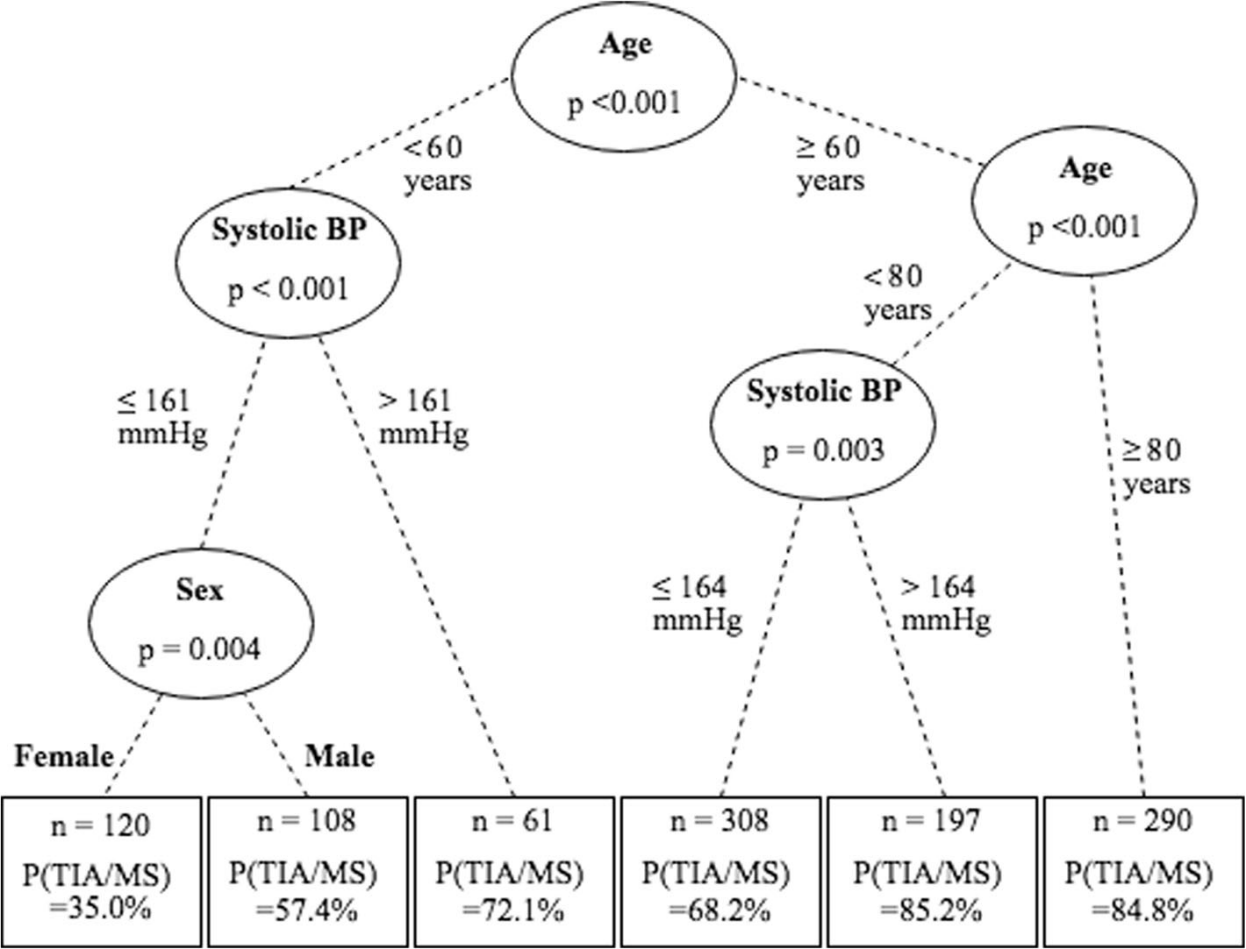
Conditional inference tree estimating the effects of age, sex, systolic blood pressure, diabetes, hypertension, and feelings of anxiety

Conditional inference trees were fit to predict binary diagnosis (TIA/MS vs. stroke-mimic) based on age (decades), sex, hypertension, diabetes, anxiety, and (i) SBP or (ii) DBP. Figure 2 shows the fitted SBP tree. The first two splits define three age-groups: < 60, 60–79, and ≥ 80 years. SBP is shown to be a useful predictor for the patients younger than 80 years, but not for those older. The tree model for DBP also first split the data on age, choosing the same cut-points as above (data not shown). We conclude that in this study, age is strongly related to TIA/MS diagnosis and that the effect of BP for predicting diagnosis may vary within the age-groups. To study the interaction of BP and age, a trichotomized age variable was used for subsequent analyses: < 60 years (141, 13.0%), 60–79 years (370, 34.1%), and ≥ 80 years (573, 52.9%). The proportion of patients diagnosed as TIA/MS in the < 60 years group is 40.4% (*n* = 57), 66.5% (*n* = 246) in the 60–79 group, and 81.2% (*n* = 465) in the ≥80 group.

### Differentiating TIA/minor stroke versus stroke-mimic

Logistic regression models were constructed with the trichotomous age variable, sex, hypertension, diabetes, anxiety, (i) SBP or (ii) DBP, and an age-BP interaction term. Reduced models that included age, sex, BP, and age-BP interaction were determined using backward elimination; see Table 2. The age-BP interaction term was significant for each model (Likelihood ratio test, SBP *p* = 0.001; DBP *p* = 0.003).

**Table 2:** Logistic regression model estimating effects of age, sex, BP, with age-BP interaction on TIA/minor stroke

Variable	*B*	*SE*	*p*	*OR*	*95% CI*
Systolic model					
Sex (male vs. female)	0.59	0.15	< 0.001	1.81	1.36–2.41
Age (< 60 vs. 60–79 years)	−0.21	1.40	0.880	0.81	0.05–12.67
Age (≥80 vs. 60–79 years)	4.06	0.97	< 0.001	58.09	8.71–387.53
Systolic^a^	0.21	0.05	< 0.001	1.23	1.11–1.35
Systolic^a^: Age (< 60 vs. 60–79 years)	− 0.04	0.10	0.671	0.96	0.80–1.16
Systolic^a^: Age (≥80 vs. 60–79 years)	− 0.21	0.06	< 0.001	0.81	0.72–0.91
(Intercept)	−2.76	0.72	< 0.001		
Diastolic model					
Sex (male vs. female)	0.59	0.15	< 0.001	1.80	1.35–2.40
Age (< 60 vs. 60–79 years)	0.84	1.37	0.542	2.31	0.15–34.17
Age (≥80 vs. 60–79 years)	4.13	0.98	< 0.001	62.13	9.03–427.61
Diastolic^a^	0.29	0.08	< 0.001	1.34	1.14–1.58
Diastolic^a^: Age (< 60 vs. 60–79 years)	−0.21	0.16	0.177	0.81	0.59–1.10
Diastolic^*^: Age (≥80 vs. 60–79 years)	−0.39	0.12	< 0.001	0.68	0.54–0.85
(Intercept)	−2.18	0.72	0.002		

*CI* confidence interval; *OR* odds ratio; *SE* standard error.

^a^Per 10 mmHg. In these models, the reference group for age is 60–79 years

**Fig. 3:**
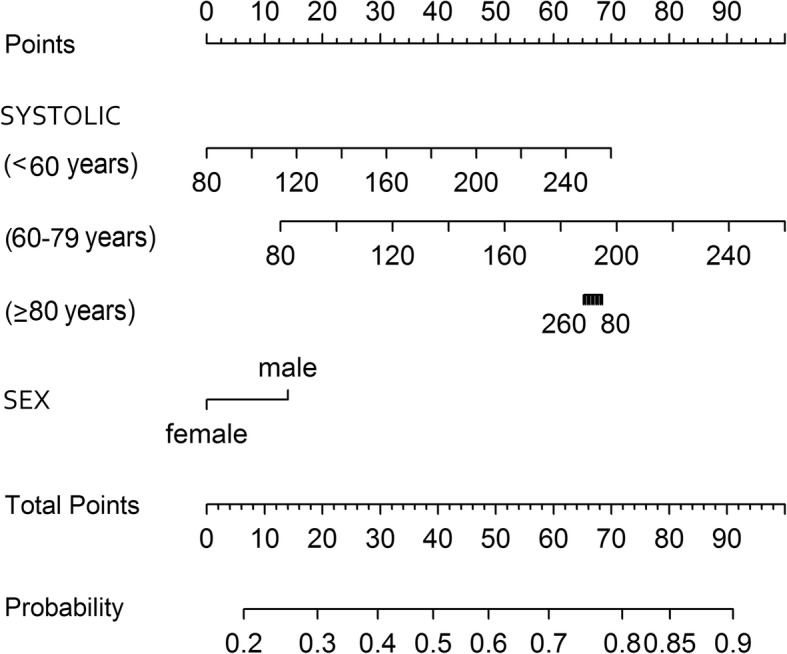
Nomogram for the reduced logistic regression model predicting TIA/minor stroke versus stroke-mimic with from sex and the interaction of age and systolic blood pressure. To use the nomogram for a patient with given SBP, age, and sex, use the ‘Points’ scale at the top of the figure to ascertain points for each variable aligning the scale vertically with the variable value. For example, for a male patient aged 50 with SBP of 120 mmHg, score 15 points for male and 15 points for age < 60 years with SBP of 120 mmHg. The ‘Total Points’ value is 30. Align the value of 30 on the ‘Total Points’ scale with the ‘Probability’ scale, to determine an approximate probability of TIA/MS of 0.40 for this patient. Note that the SBP points for patients older than 80 years decrease with increasing SBP as the SBP odds ratio for that group is less than one

From the systolic model, an increase of 10 mmHg was associated with significant increases in the odds of TIA/MS for the patients aged < 60 years (odds ratio [OR] 1.18, 95% confidence interval [CI] 1.00–1.39), and for those aged 60–79 years (OR 1.23, 95% CI 1.12–1.35), but not in the oldest patients ≥80 years (OR 0.99, 95% CI 0.92–1.07). Males showed increased odds of TIA/MS (OR 1.80, 95% CI 1.35–2.40) and compared to the intermediate group, the oldest cohort had significantly elevated odds of TIA/MS (OR 62.13, 95% CI 9.03–427.61). The nomogram for the reduced SBP model is shown in Fig. 3. This graphical representation demonstrates the usefulness of BP as a predictor for TIA/MS across the three age-groups, after adjusting for the effect of sex. See figure legend for detailed explanation of how to interpret the nomogram.

From the diastolic model, an increase of 10 mmHg was associated with a significant increase in the odds of TIA/MS for the 60–79 age-group (OR 1.34, 95% CI 1.14–1.58). The odds of TIA/MS in the other two age cohorts were not significantly affected by DBP (< 60 years: OR 1.08, 95% CI 0.83–1.42; ≥80 years: OR 0.91, 95% CI 0.78–1.07).

The internal model validation procedures for (i) SBP and (ii) DBP showed similar results. The bias-corrected AUC and Brier score were 0.699 and 0.183 for SBP, and 0.697 and 0.185 for DBP, respectively. The median number of variables selected was 4 (IQR 4–5) for both procedures. BP (by design), age, and sex were selected in all bootstrap samples, while the age-BP interaction was selected in 96.5% (systolic) and 94.5% (diastolic) of samples, suggesting these are all stable predictors. Further, the other covariates from the full model (hypertension, diabetes, and anxiety) were each selected in less than 20.5% of bootstrap models suggesting these are not stable predictors and the final model is appropriate.

## Discussion

In a detailed prospective cohort study looking at the diagnosis of TIA/MS we found that SBP measured at ED triage was significantly higher in TIA/MS patients as compared to stroke-mimics. We also found that elevated SBP increased the chance of an individual patient having had a TIA/MS; however, this was only true for patients younger than 80 years. In the patients aged < 60 and 60–79 years, SBP was associated with a significant increase in odds of TIA/MS of 18 and 23% per 10 mmHg increase, respectively, after accounting for age and sex. Elderly patients are known to have an increased risk of stroke; the results of our study suggest that having elevated SBP did not further increase this risk. In contrast, elevated SBP appears more indicative of TIA/MS in patients under 80 years old.

There are a number of possible explanations for the observed interaction between age and blood pressure in regard to stroke risk. Prior studies have observed that the prevalence of hypertension (> 140/90 mmHg) increases with age [34–37]. Analysis of the Framingham Heart Study found the prevalence of hypertension among patients ≥80 years of age to be 74% (< 60 years, 27.3%; 60–79 years, 63%; ≥ 80 years,74%) [36]. Amongst patients ≥80 years of age treated with anti-hypertensive medication, less than 50% achieve hypertension control [36, 37]. This suggests that the ability to differentiate between stroke-mimic and stroke patients on the bases of blood pressure in patients ≥80 years of age is attenuated by the lack of inter-patient variability in blood pressure values, relative to younger cohorts. Moreover, other studies [38, 39] have observed that in patients ≥85 years of age elevated blood pressure is associated with *decreased* rates of mortality, even after controlling for age and sex. Boshuizen et al. observed that only after controlling for poor health status, in addition to age and sex, that *low* blood pressure (< 140/60) was associated with increased cardiovascular and stroke mortality in patients ≥85 years of age. Comparably, Bulpitt et al. found that treatment of hypertension in patient > 80 years of age decreased stroke deaths while possibly being associated with a corresponding increase in non-stroke deaths [40]. Together, these studies suggest that for patients ≥80 years of age elevated blood pressure is of high prevalence and that the association between elevated blood pressure and patient health differs from that of younger cohorts.

Few studies have examined the importance of clinical features for distinguishing TIA/MS from stroke-mimic patients in the ED. In contrast to our finding that TIA/MS patients had significantly higher SBP at ED triage than stroke-mimics, one such study found BP on admission not to differ between TIA and stroke-mimicpatients [11]. This difference could be the result of differing TIA definitions: we used clinical plus imaging, while others use clinical-based only.

When examined by TOAST subtype, the SBP of SVO TIA/MS patients was significantly higher than stroke-mimics and every other stroke etiology individually. This corroborates Meuer’s findings [41] that, for ischemic strokes, the odds of cardioembolic versus SVO stroke increase by 20% for every 10 mmHg decrease in presenting SBP. Our results also indicated DBP to be higher in SVO patients compared with stroke-mimics, cardioembolic, cryptogenic, LAA, and competing etiologies. Another study yielded similar results in that patients with ischemic stroke of cardioembolic origin showed a significant lack of BP response on presentation compared to atherothrombotic and lacunar strokes and the lower BP in this group was associated with poor outcomes [42].

The diagnosis of TIA/MS is particularly challenging in younger patients, who have fewer of the classic risk factors for TIA/MS. For these younger patients, an early and accurate diagnosis is especially important to mitigate the risk of a recurrent, disabling stroke. Our findings could be leveraged by physicians as an indicator to further examine young patients presenting with elevated SBP in conjunction with transient neurological symptoms.

### Limitations

In general, TIA studies are challenged by the absence of a clear consensus on the definition of TIA [43] and the related issue of inter-rater variability [44, 45], making direct comparisons between studies challenging. Further, many previous studies have relied on ED discharge diagnosis [4–6, 9, 46] where accurate diagnosis is even less precise. However, these issues were mitigated in our study by the extensive imaging for both the TIA/MS and stroke-mimic groups and all cases were adjudicated by stroke neurologists.

As logistical considerations in our study mandated that the majority of patients were enrolled during daytime hours, we acknowledge a potential bias in study design. There may be a case-mix difference in TIA patients who present during daytime hours compared to patients who go to the ED at night. We were unable to investigate this possibility as we did not have a comparison sample. Additionally, as with other studies performed in the ED, BP may have been measured either sitting or supine.

## Conclusions

The inherent difficulty in diagnosing TIA/MS and distinguishing from stroke-mimic conditions in the acute setting, coupled with the need for urgent imaging for true TIA/MS patients, places an emphasis on a well-informed clinical suspicion of the illness. While imaging is useful for diagnosis, it is also an expensive, limited resource that may not be readily available. Clinical vital signs, such as BP, particularly in younger patients, could help guide the decision-making process and inform pre-imaging workup and investigations. Our findings of a statistically significant interaction between age and BP could help physicians to re-evaluate patients for a clinical suspicion of TIA/MS as part of their overall clinical gestalt for managing patients with neurological deficits in the ED. Furthermore, BP may suggest TIA/MS etiology—as in the case of small-vessel occlusion for example—which could be further used to direct patient management. These findings suggest emergency physicians may benefit from considering BP an important vital sign in younger patients that they may leverage to guide work-up and patient management.

## Data Availability

The datasets generated and/or analyzed during the current study are not publicly available in accordance with the patient privacy and confidentiality policy of Island Health.

## Abbreviations

AUC: Area under the receiver operating curve
BP: Blood pressure
CI: Confidence interval
DBP: Diastolic blood pressure
ED: Emergency departments
IQR: Interquartile range
LAA: Large-artery atherosclerosis
MS: Minor stroke
OR: Odds ratio
SBP: Systolic blood pressure
SE: Standard error
SVO: Small-vessel occlusion
TIA: Transient ischemic attack
TOAST: Trial of Org 10,172 in Acute Stroke Treatment
